# Nine months of daily LiDAR, orthophotos and MetOcean data from the eroding soft cliff coast at Happisburgh, UK

**DOI:** 10.1038/s41597-024-03499-3

**Published:** 2024-08-05

**Authors:** Catherine Pennington, Matthew Shaw, Thomas Brooks, Riccardo Briganti, Alejandro Gómez-Pazo, Gioele Ruffini, Matthew Appleton, Andres Payo

**Affiliations:** 1 British Geological Survey, Multi Hazards and Resilience, Keyworth, NG12 5GG UK; 2 ScanLAB Projects, Unit 7, 5 Durham Yard, London, E2 6QF UK; 3 University of Nottingham, Faculty of Engineering, Nottingham, NG7 2RD UK; 4 University of Santiago de Compostela, Department of Geography, Santiago de Compostela, 15701 Spain; 5 Sapienza University of Rome, Department of Civil, Building and Environmental Engineering, Rome, 00184 Italy

**Keywords:** Natural hazards, Ocean sciences, Climate sciences

## Abstract

The dynamic interaction between cliff, beach and shore-platform is key to assessing the sediment balance for coastal erosion risk assessments, but this is poorly understood. We present a dataset containing daily, 3D,colour LiDAR scans of a 450 m coastal section at Happisburgh, Norfolk, UK. This previously para-glaciated region comprises mixed sand-gravel sediments, which are less well-understood and well-studied than sandy beaches. From Apr-Dec 2019, 236 daily surveys were carried out. The dataset presented includes: survey areas, transects LiDAR scans, georeferenced orthophotos, meteorological- and oceanographical conditions during the Apr-Dec observation period. Full LiDAR point-clouds are available for 67 scans (Oct-Dec). Hourly time-series of offshore sea-state parameters (significant wave height, mean propagation direction, selected spectral periods) were obtained by downscaling the ERA5 global reanalysis data (global atmosphere, land surface and ocean waves) using the numerical model Simulating Waves Nearshore (SWAN). We indicate how to obtain hourly precipitation time-series by interpolating ERA5 data. This dataset is important for researchers understanding the interaction between cliff, beach and shore-platform in open-coast mixed-sand-gravel environments.

## Background & Summary

To better understand the evolution in time of coastal areas, remote sensing or other measurements techniques are essential^1^ and often used to correctly define the topography after a certain event^2^, to analyse the modification of the coastline over a long period of time^3^ or even to analyse land-cover/land-use changes in this very same areas^4^. This type of data are then essential to study more in depth the phenomena that cause these changes and for governments and legislators to improve planning and protection of coastal infrastructures. In this context, sediment budgets are used in coastal engineering and science studies to develop understanding of the sediment sources, sinks, transport pathways and magnitudes for a selected region of coast and within a defined period of time^5^. The sediment budget is obtained from a balance of volumes (or volume rates of change) for sediments entering (source) and leaving (sink) a selected region of coast and the resulting erosion or accretion in the coastal area under consideration. The dataset presented in this study contributes to our understanding of the poorly-known aspect of coastal sediment budgeting on soft cliff shorefaces found around the world, especially in previously para-glaciated regions^6^ and, in turn, of soft-cliff erosion and mass movement processes in glacial lithologies^7^. One of the key elements in improving this understanding is the quantification of sediment contribution to the nearshore sediment budget from cliff and shore platform back-wearing and down-wearing, respectively^8^. No studies, to our knowledge, have integrated such high-resolution, both spatially and temporally, LiDAR (Light Detection And Ranging) scans and orthophotos of a cliff-beach-platform system, with hourly meteorological and oceanographic forcing conditions, over multiple months.

**Fig. 1:**
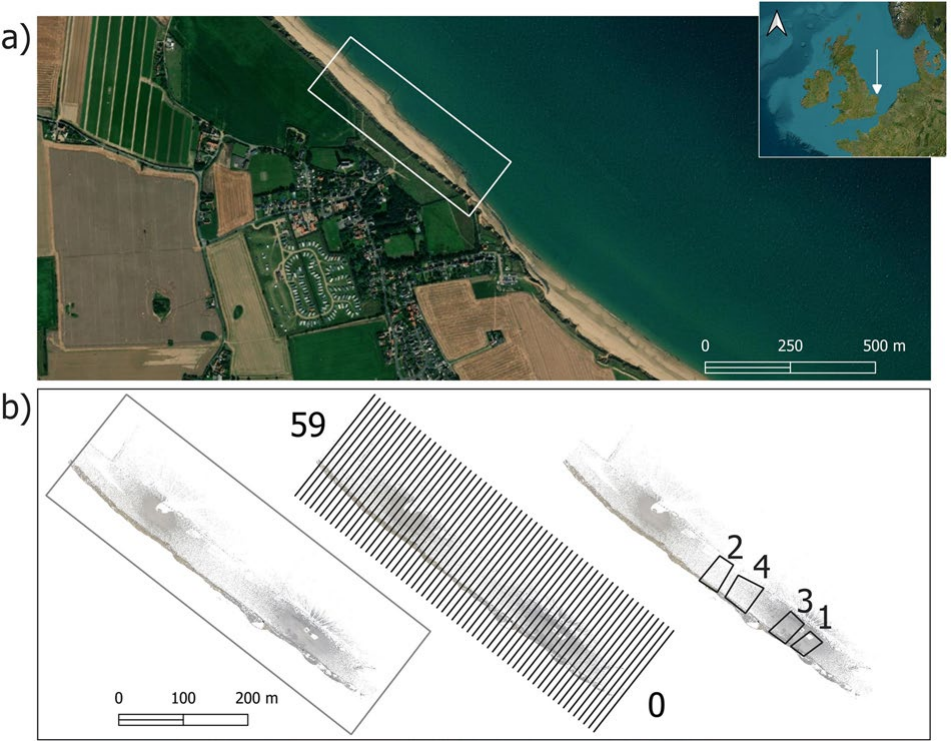
Study site location and examples of LiDAR scans outputs provided in this dataset; (**a**) The location of the study site (white arrow) in the UK and local (Happisburgh, marked as white rectangle) contexts. Map source: Esri, Maxar, Earthstar Geographics, and the GIS User Community https://services.arcgisonline.com/ArcGIS/rest/services/World_Imagery/MapServer/3; (**b**) Zenital view of the LiDAR cloud point collected on day 1 of the dataset and location of the LIDAR transects (black rectangles of 1 m width and 200 m length, numbered from 0 to 59) and areas (black polygons, numbered from 1 to 4). The density of cloud points is depicted on a grey to white scale, where grey indicates higher density. LiDAR data © ScanLAB Projects 2023 and © BGS 2023.

To fill this data gap, we present a unique dataset. This comprises daily, high-resolution, coloured LiDAR point-clouds, over a three-month window (October to December 2019), as well as transects (LiDAR point-cloud data transects), areas (LiDAR point-cloud data grids), georeferenced orthophotos and downscaled meteorological and oceanographical (MetOcean) forcing data over nine months (April to December 2019). The site is a 450-metre coastal section near the village of Happisburgh, Norfolk, on the soft sediment coast of eastern England^6^, UK (Fig. 1). We used one Terrestrial LiDAR Scanner^9^ (TLS) to obtain daily point-cloud data at two fixed locations at the study site. By using the TLS in conjunction with a high-resolution digital camera and a high-precision differential Global Navigation Satellite System (GNSS), we obtained geo-referenced, coloured point-clouds and orthophotos of the shoreface. This dataset also includes MetOcean conditions of the sea around Happisburgh, spanning the entire observation period, which have been downscaled and harmonized into hourly time series. Sea state parameters (e.g., significant wave height, mean direction of propagation, spectral periods) were obtained by using the numerical model Simulating WAves Nearshore^10^ (SWAN) to downscale the ERA5^11^ global reanalysis dataset. The sea level at the British Oceanographic Data Centre (BODC) station at Cromer, 20 km North-West of Happisburgh, was additionally used for the downscaling.

Since the full point-cloud dataset for each of the surveyed days is very large (22 GB each), smaller subsets are included with the full LiDAR dataset, hereafter referred to as either ‘areas’ or ‘transects’. The transects are 1 m wide and span the foreshore width, mimicking traditional transect-based methods for coastal monitoring. The beach areas cover smaller sections of the foreshore, situated between the two TLS scanning positions, where the point density of the cloud is large (Fig. 1b). These transects and areas allow the study of daily changes in the beach surface. The platform (an erosional rock surface underneath the beach) is usually covered by beach deposits, but periodic exposure (e.g., after storms remove the beach material) may allow study of platform down-wearing^8^. Shapefiles with the geospatial extent and geolocation of both the transects and the areas are also included in the repository.

We believe that this dataset is important for the research community interested in understanding the interactions between the cliff, beach and shore platform on similar open soft-cliff coastal environments to this study site. The high temporal frequency and spatial resolution of our dataset could allow for very accurate measurement and analysis of coastal processes affecting the Norfolk coast. Cliff collapses, changes in beach volume and morphology, and platform down-wearing are evident from initial investigations into the scans; the dataset could develop understanding of the causes and effects of these processes.

## Methods

### Study site

The cliffs at Happisburgh are between 6 and 10 m high and underlain by the Wroxham Crag Formation (brown sands and clays with gravel seams) that is typically buried beneath modern beach material but periodically exposed following storms^7^. Overlying this, is a sequence of tills, separated by beds of stratified silt, clay and sand; these include the Happisburgh Till, Ostend Clay and Happisburgh Sand Members which make up the cliff^7^. Sea levels at the study site have been rising for millennia^12^, and under natural conditions, the coast of Norfolk is erosional^8^. In response to the 1953 flooding, a continuous line of defences was constructed in the 1960s to protect the village of Happisburgh, extending 15 km Northwest to Trimingham. These comprised sheet piles, crested with a sloping timber palisade, fronted by groynes. The design was intended to reduce cliff recession rather than entirely prevent it, and to allow some sediment transport to sustain the beaches. Since 1996, the Environment Agency (EA) has undertaken a series of beach nourishments (around 150,000 m^3^/yr on average^13^) at Sea Palling, 5 km to the South and down-drift of Happisburgh. The nourishment scheme aims to offset the reduction in sediment supply from cliff erosion along the Happisburgh–Trimingham coastal section. The beach nourishment also aims to protect a sea wall at Sea Palling, through the formation of a beach which can dissipate wave energy, especially in storm conditions. Over time, deterioration of the Happisburgh coastal defences due to wave action has led to their failure. This led to the rapid formation of an embayment to the southeast of our study site^6^. The EA^14^ report that, following structure failure, up to 140 m of recession occurred within the Happisburgh embayment between 1992 and 2012. During the data collection for this work, the sheet pile, palisades and groynes at the study site are still present but severely damaged and in poor condition with sections now completely removed.

The site is fully exposed^7^ to Southern North Sea waves, with average annual significant wave heights (H_s_) of 0.9 m and peak periods (T_p_) of 4 s from the N-NNE. The wave climate is non-seasonal with similar moderate-energy summers (July to September, H_s_ = 0.95 m and T_p_ = 4 s) and moderate-energy winters (October to June, H_s_ = 0.92 m and T_p_ = 4 s), and extreme wave heights exceeding H_s_ = 6 m and T_p_ of 10 s. The coast is macrotidal, with a mean spring range of 4.23 m and mean neap range of 2.09 m, obtained from the observed and predicted tidal elevations at the nearest tidal gauge station at Cromer during the years 2008 to 2026 (http://www.ntslf.org/tides/hilo). The sea around the South-East of England is particularly vulnerable to storm surges. The 10 largest^11^ skew surges (the difference between the maximum observed sea level and the maximum tide) registered at Cromer varied from 1.13 to 1.76 m and all occurred between November and February.

### Experimental setup used for the daily TLS surveys and orthophotos

**Fig. 2:**
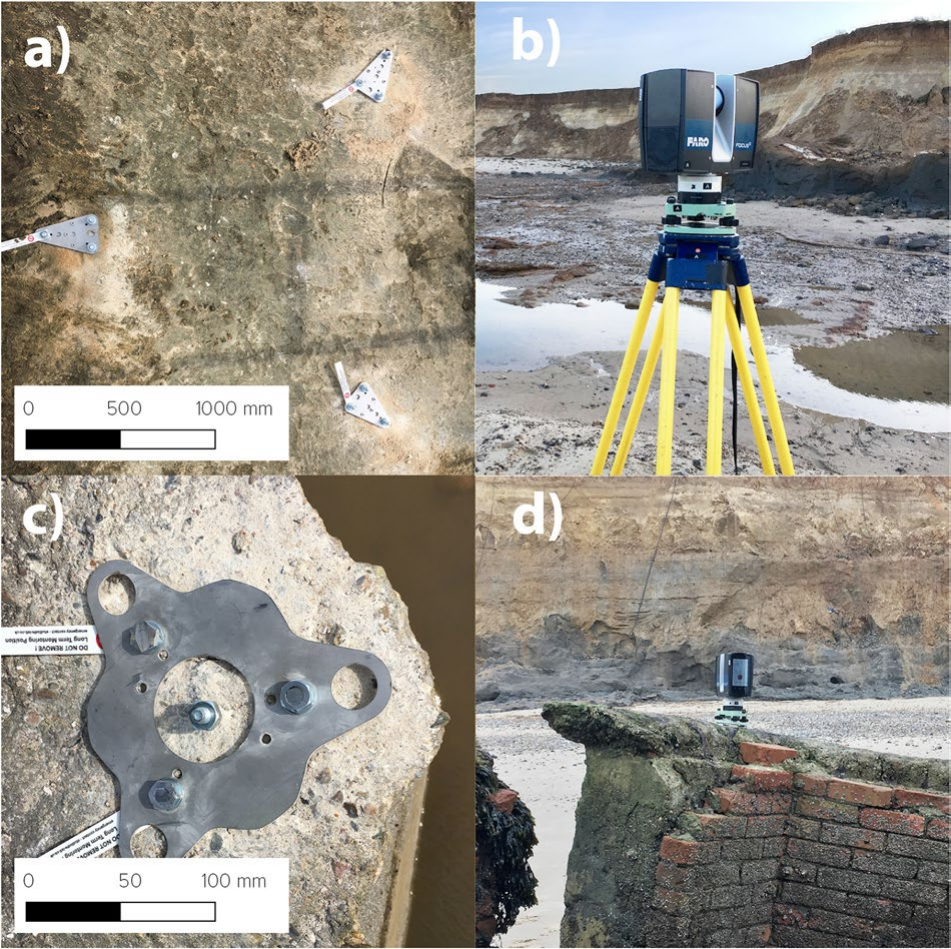
The experimental setup of the TLS used at Happisburgh. In panels: (**a**) The permanent scanner position on the staircase (location 1). When the scanner is attached, each leg of the tripod connects to the steel elements, and tripod leg lengths are kept equal between scans; (**b**) The TLS fully set up at the staircase (location 1); (**c**) The permanent tribrach attached to the pillbox (location 2) allows for consistent scanner positioning; (**d**) The TLS set up on the pillbox (location 2). Photographs © ScanLAB Projects 2023.

Carrying out TLS surveys in dynamic coastal environments is challenging due to a lack of permanent reference points as all physical natural surfaces (i.e. beach, cliff) are subject to rapid and continuous change. Figure 2 shows the experimental LiDAR setup used to obtain the point-clouds. The daily TLS surveys used a single FARO S350 LiDAR scanner placed at two fixed locations spaced 178 m apart, around 40 m from the base of the cliff. These fixed locations were intentionally chosen as they were the objects in the landscape least likely to move as a result of coastal processes and the repeated GPS surveys of these points showed no movement in the survey period; one location was on the concrete foundation of what was previously a staircase (location 1), the other was on a partially buried concrete pillbox (location 2). The pillbox was constructed on the cliff top in 1940 but, due to cliff recession, is now on the beach, 40 m from the cliff toe. To secure the TLS to the staircase, a tripod was used, and the leg lengths were kept fixed throughout the entire observation period. However, for the pillbox, a stainless-steel element was attached to the concrete, providing a stable anchoring point. Both locations were accessible during low tide and were sufficiently stable for reliable co-registration (the technique used to integrate multi-angle, multi-temporal remote sensing data) over the observation period. The TLS surveys were aligned with the horizon to assist the co-registration process as detailed below.

The duration of scanning at each location was around 30 minutes, with a resolution of 40960 × 17067 (max 699.1 M points). The TLS was moved from one location to the other consecutively. The TLS had a maximum usable target distance of 350 m and point distance of 1.5 mm at 10 m. The TLS data were exported with a maximum distance filter of 610 m for the location 2 and 410 m for the location 1. The TLS beam divergence was 0.3 mrad, resulting in a beam diameter at the cliff and beach surface ranging from 9 mm to 100 mm and 1.2 mm to 100 mm respectively. This was calculated on the scan taken from location 1 on 23^rd^ Dec 2019 (the scan position was 4 m above the beach surface and 30 m from the cliff).

The raw data collected were filtered and co-registered during post-processing using FARO^®^ Scene Software 2019.0.1.1653. The global position of each TLS survey was recorded using a Leica GS15 at the end of data capture period. Then the exported scan data were transformed spatially to the GPS position and orientation; the zero elevation was shifted to be Ordnance Datum Newlyn^15^ using ScanLAB’s proprietary processing software.

To create coloured point-clouds, a high-resolution digital camera (Nikon D7000 DSLR with a Nikon 10.5 mm AF Fisheye lens) was used in conjunction with the TLS to capture red-green-blue (RGB) colour data of each scan. The images captured were 16.8 MP NEF format. Colour temperature was captured at 4550 K for every image, exposure was adjusted as necessary based on the light conditions each day. The camera was positioned with its lens centred at the mirror position of the TLS and rotated around this point using a Nodal Ninja^16^ panoramic tripod head. The camera captured 36 images per scan to create a 360° panoramic image. These were combined with the TLS scans in the post-processing phase. There were 12 angles captured per panorama, 6 × 60° horizontal intervals and 2× vertical intervals at −10° and +25°, each with a set of bracketed exposures (3 images taken at −1 ev, +0 ev and +1 ev relative to the required exposure at the moment of capture). The images were combined into 360° high dynamic range (HDR) panoramic images using PTGUI 11 software. These images are in the format of 8-bit PNGs with a resolution of around 14000 × 7000 pixels. The panoramic images were then adjusted in Adobe Lightroom 2019, primarily to balance the exposure and colour-temperature over the set of panoramas. These panoramic images were then projected onto the point-cloud data as described in the Technical Validation section.

**Fig. 3:**
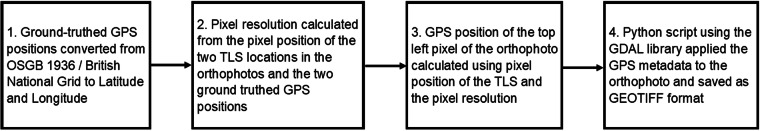
Workflow georeferencing orthophotos using python’s GDAL library.

The orthophotos are point-cloud renders of the scan data created using ScanLAB’s proprietary point-cloud rendering engine: (1) colour orthophotos are rendered using the colour information projected onto the scan during post-process colourisation, (2) intensity orthophotos are rendered using the intensity data for each scan. The orthophotos are rendered using an orthographic virtual camera which frames the useful extents of the scan data and is orientated such that the rendered orthophoto is “north-up”. Finally, the orthophotos are georeferenced using python’s GDAL library and ground-truthed GPS measurements taken at the two TLS positions on site. This procedure was carried out as in Fig. 3.

### Modelling methodology used to downscale the MetOcean forcing conditions for the study period

**Fig. 4:**
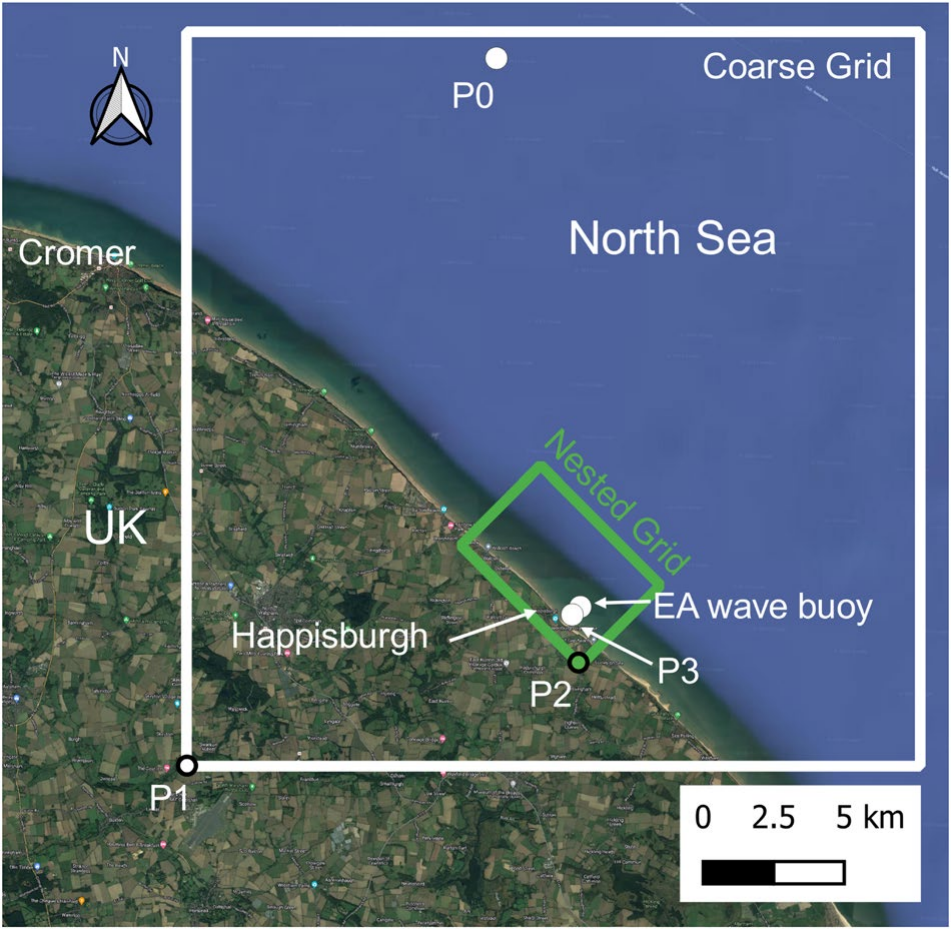
Position and extent of the two computational domains used for the SWAN computation. Notable points for the computation and validation of the MetOcean data and the location for which the precipitation data are obtained (P3) are also indicated. Map Data: Google ©2023/SIO, NOAA, U.S. Navy, NGS, GEBCO. Image generated by University of Nottingham.

The numerical model SWAN^10^ (v41.31a; https://swanmodel.sourceforge.io) was used to simulate hourly sea states at the study site. The model was run in non-stationary mode. Two numerical domains were used to compute the sea state characteristics: a coarse domain (26 km × 26 km; grid spacing 100 m), and a fine domain (4 km × 6 km; grid spacing 10 m), nested in the coarse one, rotated 45° counter-clockwise to capture the orientation of the coastline and, in turn, the orientation of the beach LiDAR transects (Fig. 4). The coordinates of the Southwest corners of the coarse and fine domains, indicated as P1 and P2, respectively, are shown in Table 1 and Fig. 4. For the coarse domain, the boundary conditions were uniform along the north, east and west boundaries, and equal to the wave conditions (H_s_, T_p_ and mean wave direction) at the ERA5 node indicated as P0 in Table 1 and Fig. 4. The wind speed on both domains was considered uniform and provided using hourly time series obtained from ERA5 reanalysis at P0. All parameters from ERA5 reanalysis were obtained by using the bilinear interpolation implemented in the Copernicus/ European Centre for Medium-Range Weather Forecasts (ECMWF) data manager. The bathymetry used was obtained from the OceanWise 1 Arc second Digital Elevation Model (DEM; https://maps.oceanwise.eu/).

**Table 1:** Easting and Northing coordinates in OSGB 1936/British National Grid, EPSG code: 27700 of notable points for the MetOcean data.

Point	Coordinate	
	Easting (m)	Northing (m)
P0	399337.60	5873322.97
P1	388354.00	5848262.00
P2	402318.00	5851840.00
EA wave buoy	402308.86	5853905.30
P3	402021.02	5853627.03

The precipitation time series can be obtained by downloading the ERA5 reanalysis also using the bilinear interpolation implemented in the Copernicus/ECMWF data manager. Figure 1 and Table 1 show the location and coordinates of the interpolation point (P3) and the Environment Agency (EA) wave buoy used to validate the wave simulations.

## Data Records

### Data repository

These datasets are available on a public data server at the Centre for Environmental Data Analysis (CEDA) repository^17^ funded by the Science and Technology Faculties Council (STFC) and the Natural Environment Research Council (NERC). They are accessible: 10.5285/2c6f3201f01d4346a97ff8f08a8c15c9 and individually at:

1. (October-December 2019) Point-cloud XYZ LiDAR data^18^. BGS © UKRI and ScanLAB Projects Ltd 2023. 10.5285/b8cf940850164ebeb4cba343384f88b8
  Released under a Creative Commons Attribution 4.0 International (CC BY 4.0) licence. Custodians are Catherine Pennington (British Geological Survey) and Matthew Shaw (ScanLAB Projects Ltd).
2. Transects and Areas LiDAR data and shape files (06/04/2019-23/12/2019)^19^, BGS © UKRI 2023. 10.5285/11e55bd0-782d-5013-e063-6c86abc043bb
  Released under a Creative Commons Attribution 4.0 International (CC BY 4.0) licence.
3. Modelled Nearshore Wave Conditions, Happisburgh, UK, (23/03/2019-31/12/2019)^20^, BGS © UKRI 2023. 10.5285/14dd5580eab9410fb3696340711b1d67
  Released under a Creative Commons Attribution 4.0 International (CC BY 4.0) licence.
4. Daily Colour and Intensity Orthophotos of the Cliff and Beach at Happisburgh, Norfolk, UK. (06/04/2019-23/12/2019)^21^. BGS © UKRI and ScanLAB Projects Ltd 2023. 10.5285/1159923a-e983-2bb1-e063-6c86abc061b9

Released under a Creative Commons Attribution 4.0 International (CC BY 4.0) licence.

### Data file format

The TLS point-clouds are provided as ASCII (YYYY-MM-DD.xyz) files, where YYYY, MM and DD are the year, month and date of the survey. The filenames for the sub-sampled point-cloud data for the 60 transects and 4 beach areas have an additional letter followed by two digits after the date (YYYY-MM-DD-XNN.xyz) where X is B for beach areas and T for transects and NN indicates the beach/transect number. The spatial extent of the beach areas and transects, marked on Fig. 1 are provided as ESRI shapefiles (Grid-BNN.shp and Grid-TNN.shp) where NN indicates the Beach area/Transect number.

This database also includes two types of georeferenced images (colour and intensity) in TIFF format with filename YYYY-MM-DD_***_TYPE_***, of 5760 × 3240 pixel resolution. YYYY-MM-DD indicates the capture date of the corresponding scan.

The wave data are provided as.mat files. A Microsoft Windows executable app file is included in the repository to convert the.mat files into.csv files containing the daily wave data. The filename format of the converted files is: <variable name>_YYYYMMDDHHMMSS.csv. A full list of the time series generated can be found in Table 2. The Universal Transverse Mercator (UTM) coordinates of each data point can also be exported. The precipitation time series at the study location and the sea level at Cromer are provided as individual.csv files. The precipitation data are provided in two-column.csv files, with the first column representing time, and the second being precipitation.

**Table 2:** Information on variables stored in data files.

Band/Column/Definition			Variable	Units
Georeferenced Orthophotos (*.tif) Resolution: 5760 × 3240 pixels				
Band 1			Red	0–255
Band 2			Green	0–255
Band 3			Blue	0–255
MetOcean (*.csv)				
Time			Time	Hours
Grid point X coordinate			Xp	Easting (m)
Grid point Y coordinate			Yp	Northing (m)
Significant wave height			HSig	m
Peak Period			RTpeak	s
Spectral period m01			Tm01	s
Spectral period m02 (mean zero crossing period)			Tm02	s
Spectral period m-10			Tm_10	s
Mean direction of propagation			Dir	Degrees from North
**Column Number**	**Variable Name**	**Definition**	**Unit**	**Range**
Point-Cloud/Beach/Transect (*.xyz)				
1	X	Easting	m	—
2	Y	Northing	m	—
3	Z	Elevation	m	—
4	R	Red	—	0–255
5	G	Green	—	0–255
6	B	Blue	—	0–255
7	I	Intensity	—	0–255
Wave Climate Time Series (*.nc)				
—	Botlev	Bottom Level	m	- 47.96 - 0.00 (4sf)
—	Depth	Water Depth	m	—
—	Dir	Mean Wave Direction	degrees	0–360
—	Dissip	Energy Dissipation	m²/s	—
—	Dspr	Directional Spreading	degrees	—
—	Hsig	Significant Wave Height	m	—
—	Qb	Breaking Wave Fraction	—	0–1
—	RTpeak	Relative Peak Period	s	—
—	Tm01	Mean Absolute Wave Period	s	—
—	Tm02	Mean Absolute Zero Crossing Period	s	—
—	Tm10	Energy Period	s	—
—	Xp	Grid Points (Easting)	m	388353–414353
—	Yp	Grid Points (Northing)	m	5848262–5874262

The location of the transects and areas are provided as shape files with the filenames PlatformTransects.zip and PlatformAreas.zip. This zip files contains all the shapefiles representing the extent of the transects and areas using the coordinate reference system EPSG:27700 - OSGB36/British National Grid and meters as unit. This shape files only contains one attribute that correspond with the feature identification or fid as shown in Fig. 1.

### File structure

The data structure for this database is summarized in Table 2. The point-cloud files have seven columns (x, y, z, r, g, b, i), where the first three columns correspond to the georeferenced position values (x, y) and the elevation (z). The next three columns correspond to the RGB colours and the final column represents the intensity.

### Data temporal coverage

The coastal environment presented practical challenges for the data collection team. For safety reasons, TLS positions were only accessible at low tide. There were days when the team were prohibited from accessing survey locations due to adverse weather conditions, when there was insufficient overlap between daylight hours and low tide, or when the water level at the highest of low tides meant it was not enough time to safely carry out a survey.

**Fig. 5:**
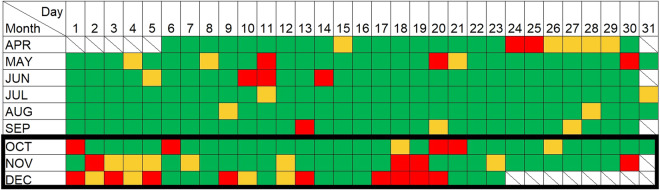
TLS survey temporal coverage. Green represents days where scans were performed at both anchors; orange represents a single scan, and red represents no scans. Diagonal lines mark days outside of the analysed period. Contains scheduling data © ScanLAB Projects 2023. The black box indicates the availability for the entire LiDAR point cloud dataset, transects and areas datasets and orthophotos are available for the entire survey temporal coverage.

Figure 5 illustrates the temporal coverage of the TLS surveys for the full 262 days observation period. A full TLS survey, involving scans performed at both locations 1 and 2, was possible for around 80% of the study period. For around 10% of the study period, scans were performed at only one of the anchor locations. The remaining 10% accounts for when surveys were not possible, resulting in data collection occurring on 236 of the 262 study days. Exact dates are indicated on Fig. 5.

### Data visualizations

There are many methods that can be used to visualise point-cloud data^9^. We have used ScanLAB Proprietary Processing and Rendering Software to create 3D views of the daily data.

## Technical Validation

### Point-cloud data

The point-cloud data contained in this database have undergone the technical validation and noise removal processes outlined below:

1. Filtering: Point-cloud data have been filtered in Faro® Scene Software using the following filter settings to remove erroneous data from the scans; Dark Point Filter: 300, Stray Point Filter Threshold: 10%. A Dark Scan Point Filter with a threshold of 300 removes scan points with a low reflective return value which are unlikely to have reflected off true or reliable surfaces. Stray Point Filters with a threshold of 10% removes scan points that result from hitting two objects with a thickness of the laser beam or by hitting no object at all, for example the sky.
2. Co-registration: This was performed in Faro® Scene Software in two stages. Firstly, for each TLS location the series of daily scans were registered together by manually selecting physical reference points between the scans to perform Target Based Registration and by manual angular adjustments. This process took place independently of the other TLS location, resulting in a set of co-registered daily TLS surveys for each location. Secondly, these sets of co-registered daily TLS surveys were then aligned to each other by manually selecting reference points between the scans from each location. The stability of the anchor at location 1 relative to that at location 2 was verified using scan data measurements from multiple days throughout the observation period with measurements found to be consistent.
3. Colouring: The projection of the HDR colour panoramas onto the scan was achieved using a ScanLAB proprietary plugin for Faro® Scene Software. This aligned the HDR colour panorama accurately to the colour image captured by each TLS survey.

### Downscaled wave data

SWAN results were validated against the wave measurements of the EA wave buoy (Fig. 4). To measure the accuracy of the model prediction, the bias, RMSE (Root Mean Square Error) and the scatter index (SI) for the significant wave height, peak period, and the mean wave direction are calculated.

For a general timeseries of observations *o*_*i*_, and a corresponding time series of predictions *m*_*i*_, where *i* = 1, …*N*, and *N* is the number of observations, the bias, RMSE and SI can be calculated using the following formulae:

1 $$bias=\frac{1}{N}{\sum }_{i=1}^{N}{m}_{i}-\frac{1}{N}{\sum }_{i=1}^{N}{o}_{i}$$

2 $$RMSE=\sqrt{\frac{1}{N}{\sum }_{i=1}^{N}{\left({m}_{i}-{o}_{i}\right)}^{2}}$$

3 $$SI=\frac{RMSE}{\frac{1}{N}{\sum }_{i=1}^{N}{m}_{i}}$$

The computed bias, RMSE and SI are shown in Table 3.

**Table 3:** BIAS, RMSE and SI results for comparison of modelled significant wave height (*H*_*s*_), spectral period (*T*_*m02*_) and mean wave direction (*D*_*m*_) with buoy observations.

	Hs	T_m02_	D_m_
bias	−0.0362 m	−0.5827 s	−0.4249 °**N**
RMSE	0.1714 m	2.3310 s	1.1796 °**N**
SI	0.2666	0.3672	0.2872

**Fig. 6:**
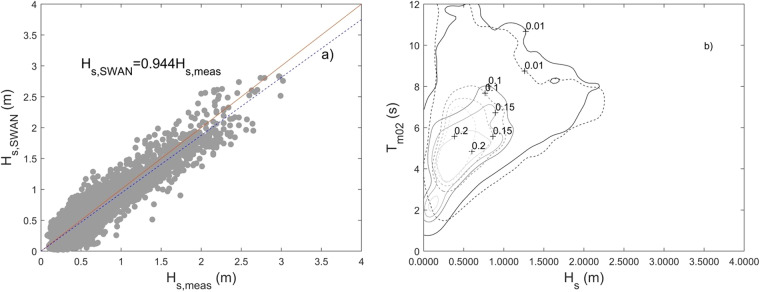
(**a**) H_s_ modelled (H_s,SWAN_) vs H_s_ measured (H_s,meas_), gray markers, samples, red line, perfect fit line, blue line, best fit line (equation indicated in the panel. (**b**) Joint distribution of H_s_ and T_m02_, dashed lines: empirical joint probability density isolines for the observations, continuous lines: corresponding isolines for the model. Contains Wave Buoy data provided by Channel Coastal Observatory on behalf of © the Environment Agency and the Anglian Coastal Monitoring Programme (https://wavenet.cefas.co.uk/Map).

Figure 6a shows the comparison between H_s_ modelled (H_s,SWAN_) and H_s_ measured (H_s,meas_) at the EA buoy, with the best fitting linear relationship to account for the bias. Figure 6b shows the modelled and observed empirical joint probability distribution of H_s_ and T_m02_. The maximum difference between the two is seen for the empirical joint probability larger than 0.15, corresponding to the peak of the joint distribution.

## Usage Notes

All point-cloud data are referenced in a projected coordinate system (OSGB 1936/British National Grid, EPSG code: 27700). The elevations are relative to the Ordnance Survey Newlyn Datum^13^. All datasets listed in Data Records section are freely available in a public repository with a Creative Commons CC BY 4.0 license.

To provide guidance on data storage requirements, the following applies:

- Point-cloud XYZ files are 21-22 GB. The transects/areas have a much smaller file size. The maximum size for these is 40 MB, but most transect/areas are much smaller. The maximum size for the areas is around 3 GB.
- TIFF images are 55MB
- For wave data: nested domain averages at 2.2 GB, coarse domain average 640 MB.

A full list of the software used to generate the point-cloud dataset are below:

- FARO® Scene 2019 + ScanLAB Proprietary Panorama to Scan Projection Plugin
- PTGui 11
- Lightroom 2019
- ScanLAB Proprietary Processing and Rendering Software
- Rhino 5 + Grasshopper 0.9.0076

For the downscaling of the ERA5 wave data we have used SWAN version 41.31a.

The authors recommend the use of Nubigon version 6.0.0 or ArcGIS Desktop (ArcMap) 10.8.2 software for visualising LiDAR data.

## Data Availability

The precipitation time series at the scan locations and sea state parameters at P0 can be downloaded from the Climate Data Store (CDS) using the Python scripts available at the Nottingham Research Repository (10.17639/nott.7308)^22^. The scripts require the installation of the CDS Application Programme Interface (API). Guidance is available at https://cds.climate.copernicus.eu/api-how-to. The provided MetOcean data can be visualised and/or analysed using standard releases of any commercial and open- source scientific software/language (such as MATLAB and Python) that can read NetCDF files.
